# Defining and utilizing individualized learning objectives to achieve learning priorities for global health leaders

**DOI:** 10.1371/journal.pone.0270465

**Published:** 2022-06-28

**Authors:** Meike Schleiff, Elizabeth Hahn, Caroline Dolive, Lillian James, Melanie Atwell, Bhakti Hansoti

**Affiliations:** 1 Department of International Health, Johns Hopkins Bloomberg School of Public, Baltimore, Maryland, United States of America; 2 Sustaining Technical and Analytical Resources (STAR) project, Public Health Institute, Washington, DC, United States of America; 3 Department of Emergency Medicine, Johns Hopkins School of Medicine, Baltimore, Maryland, United States of America; Harvard Medical School, UNITED STATES

## Abstract

**Introduction:**

Learning objectives (LOs) are a common tool used to define learning goals and guide curricula. As the field of global health has expanded, more rigorous and tailored approaches to effectively teach the next generation of the workforce are needed. The STAR project developed and utilized individualized LOs as the basis for on-the-job learning plans for senior global health leaders from low- and middle-income countries and from the US.

**Methods:**

We analyzed basic demographic information and LOs from 36 STAR fellows. Descriptive statistics provided an overview of the STAR fellows, competency areas and planned outputs of their LOs. We utilized qualitative thematic analysis to further explore the LOs themselves.

**Results:**

STAR fellows were based in the US and in low- and middle-income countries (LMICs). The majority had over 10 years of experience and at least one advanced degree. Fellows commonly worked on LOs related to capacity strengthening, communications, and development practice. Capacity strengthening LOs focused on mentorship, decision-making, and technical skills such as data analysis. Communications LOs focused on language skills, dissemination of information, and writing. Development practice LOs included gaining understanding of key stakeholders in global health and building effective partnerships and teams.

**Discussion:**

Our experience developing tailored LOs provided deeper understanding of diverse learning needs of global health leaders. While not representative of all global health learners, we captured priorities of senior US- and LMIC-based leaders and identified common themes for learning. Despite the labor required to tailor curricula in this way, more global health education programs can benefit by integrating similar processes.

## Introduction

Learning objectives (LO) are a critical tool commonly used to define learning outcomes and focus teaching. They help to clarify, organize and prioritize the learning needs for the learner and are utilized in both the development of individualized learning plans and to guide curriculum development [1]. Traditionally learning objectives include an action verb and state specifically who will do what by when [1]. In order to accommodate a wide range of learning styles and an array of learners, Bloom’s Taxonomy is often used to guide the kind of learning activities needed to deliver appropriate content and experiences to learners [2]. Learning objectives may be defined at the level of a degree program, course, or particular assignment or lecture, though they are generally defined for a specific course or tailored training program and describe what a learner will be able to do after completing that program [3–5]. Conversely, competencies are usually defined for an entire curriculum or for a particular job description and include knowledge, skills, and attitudes that will enable an individual to master the job or achieve the minimum requirements for the curriculum completion [6, 7].

Global health training opportunities have expanded dramatically in the past few decades [8–12]. Pedagogical approaches to supporting emerging leaders in this field have begun to take shape, but more experience and further evidence and documentation of strategies and outcomes of these programs are needed [7, 10, 13–16]. In recent years there has been an increasing focus on building the global health workforce, with the delivery of technical and leadership focused training programs [12, 17, 18]. In order to deliver effective programs, it is necessary to understand the specific needs of the global health workforce, where training effort should be directed, and how existing curricula can be strengthened. A synthesis of individual learning priorities and specific activities based on an analysis of individualized learning objectives is one such approach to meet this need.

The Sustaining Technical and Analytic Resources (STAR) project aims to provide tailored learning opportunities to support career progression and job performance to senior global health fellows working with USAID headquarters as well as within country-level missions in low- and middle-income countries (LMICs) [16]. Roles and job responsibilities differ based on the level of the position (mid-career vs. senior) and placement (at USAID, the sponsor, or at a USAID-recipient such as a Ministry of Health). Fellows working in the US are US citizens, while those working in LMICs are native to the country in which they work or are from another country in the same region. These fellows often have many years of experience and the majority hold graduate degrees in public health and related fields. Individual learning objectives are developed as each fellow is onboarded to the STAR project based on an assessment of their prior global health experience and skills and their career goals [16]. Given the diversity of backgrounds and experiences and roles that the fellows are bringing to this project, being able to tailor learning to their needs rather than developing one uniform curriculum was a priority identified by all of the key stakeholder in this project, including the learners themselves. The tailored learning objectives then guide the development of an individualized learning plan (ILP) that includes activities geared towards the achievement of the learning objectives over the course the two-year fellowship.

In this paper we seek to provide a comprehensive synthesis of the learning objectives developed for STAR fellows to better understand the learning needs of mid-career and senior global health professionals.

## Materials and methods

We included 36 STAR fellows in this analysis, which consisted of all fellows who had completed the project onboarding process as of June 26, 2020. Details of the overall learning curriculum for STAR fellows, the competency framework, and training plans are provide elsewhere [16]. All fellows’ underwent an onboarding process which included a competency assessment and a series of discussions with the STAR learning team to develop and refine a set of individualized learning objectives. All participants consented to the use of the programmatic data as part of this research study.

Data utilized included 1) participant profile data from the project’s online participant database, including their position title and background information, and their technical area of focus, and 2) individualized learning objectives. The study team included members of the STAR learning team and additional graduate students from Johns Hopkins School of Public Health, who contributed to the data analysis and manuscript development.

Quantitative analysis was completed using Stata v.15 (StataCorp, LLC, College Station, TX, USA). A descriptive statistical approach was used to provide an overview of the characteristics of the fellows, the learning objectives selected for STAR fellows, and the anticipated outcomes associated with the specific learning objectives.

Each learning objective underwent qualitative thematic analysis. We created deductive codes that included the STAR competency domains [16], Bloom’s Taxonomy levels(2), and an inductive set of skill and knowledge outcomes as well as products that fellows would deliver upon successfully completing the activities related to a particular learning objective. We coded the learning objectives in Dedoose © and added additional codes inductively to the codebook as needed.

This study was reviewed and approved by the IRB of the Johns Hopkins Bloomberg School of Public Health (IRB00011259) and by the IRB of the Public Health Institute (IRB #I19-022).

## Results

### Overview of participants and focus of learning objectives

Overall, 36 STAR fellows were included in this analysis, of which 17 were US-based and 19 were LMIC-based fellows. The majority of US-based fellows were female (10, 58%) versus more males (15, 79%) in LMICs (Table 1). The majority of fellows had greater than or equal to 10 years of field-based experience (US: 13, 76%, LMIC: 16, 84%). More than half (9, 52%) of US-based fellows held doctoral degrees while seven (38%) of LMIC-based fellows held doctoral degrees.

**Table 1:** Characteristics and main areas of focus for US-based versus LMIC-based fellows.

	US-based Fellows	LMIC-based Fellows	Total
	(n = 17)	(n = 19)	(N = 36)
Gender			
*Male*	7 (41.2%)	15 (78.9%)	22 (61.1%)
*Female*	10 (58.8%)	4 (21.1%)	14 (38.9%)
Previous Professional Experience			
*<10 years*	4 (23.5%)	3 (15.8%)	7 (19.4%)
*≥10 years*	13 (76.5%)	16 (84.2%)	29 (80.6%)
Highest Education Attained			
*Bachelor*	1 (5.9%)	3 (16.7%)	4 (11.4%)
*Master*	7 (41.2%)	8 (44.4%)	17 (44.4%)
*Doctoral*	9 (52.9%)	7 (38.9%)	16 (44.4%)

The STAR Project developed a competency framework consisting of eight core competency domains and eight technical and ten content skill areas to structure learning activities (S1 Annex). Both US-based and LMIC-based fellows most commonly focused on three core competencies: capacity strengthening, communication and interpersonal effectiveness, and development practice (Table 2). A set of tables each set of competencies and their frequency associated with what kind of learning output was planned for each LO can be found in S2 Annex. These same three core competencies were the most likely to have a specific output defined in the LO; common outputs included that the fellow would have mentored or supervised others, advised a program, or developed communication materials.

**Table 2:** Top 3 competencies selected in learning objectives for US-based versus LMIC-based fellows.

	Learning objectives for US-based Fellows	Learning objectives for LMIC-based Fellows	Total
	(n = 60)	(n = 63)	(N = 123)
**Core competencies**			
* Capacity Strengthening*	22 (36.7%)	25 (39.7%)	47 (38.2%)
* Communication & Interpersonal Effectiveness*	21 (35.0%)	14 (22.2%)	35 (28.5%)
* Development Practice*	10 (16.7%)	10 (15.9%)	20 (16.3%)
**Skill Competencies**			
* Data Analysis & Biostatistics*	10 (16.7%)	13 (20.6%)	23 (18.7%)
* Data Science & Informatics*	10 (16.7%)	5 (7.9%)	15 (12.2%)
* Monitoring*, *Evaluation*, *and Learning*	6 (10.0%)	8 (12.7%)	14 (11.4%)
* Supply Chain*	0 (0.0%)	12 (19.0%)	12 (9.8%)
**Content Competencies**			
* Infectious Diseases*	18 (30.0%)	36 (57.1%)	54 (43.9%)
* HIV*	4 (6.7%)	7 (11.1%)	11 (8.9%)
* Nutrition*	3 (5.0%)	1 (1.6%)	4 (3.2%)

The top three content areas of focus for the LOs of US-based fellows included data science and informatics (n = 10, 17%), data analysis and biostatistics (n = 10, 17%), and monitoring, evaluation, and learning (n = 6, 10%). For LMIC-based fellows, the most common skill foci of LOs included data analysis and biostatistics (n = 13, 21%), supply chain management (n = 12, 19%), and monitoring, evaluation, and learning (n = 8, 13%). Common skill LO outputs included advising, development of analytical tools, and programs reports and data visualizations (S2 Annex). Most fellows worked within the field of infectious diseases (including TB but not HIV) with fellows focused on HIV (US-based: 4, 6.7%; LMIC-based: 7, 11.1%) and nutrition (US-based: 3, 5%; LMIC-based: 1, 1.6%) a close second and third. The common outputs for infectious disease LOs were advising and development of an analytical tool (S2 Annex).

### Type of learning objective based on seniority

**Fig 1:**
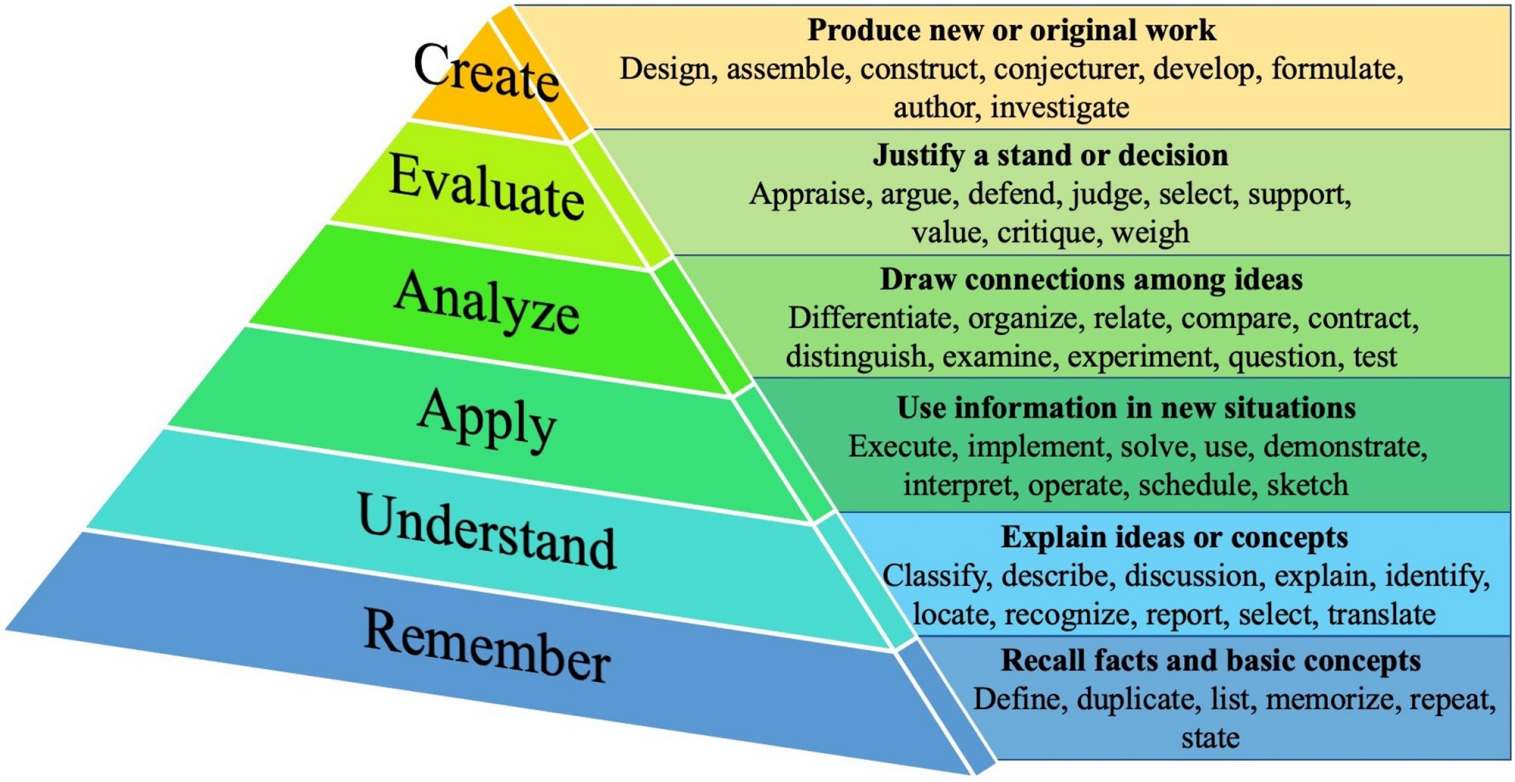
Levels and associated verbs for LOs in Bloom’s Taxonomy.

Bloom’s Taxonomy outlines six levels of knowledge comprehension and utilization from remembering and understanding at the basic levels, to applying and analyzing concepts in the middle, and to evaluating and creating new works at the highest levels (Fig 1) [2].

The level of Bloom’s Taxonomy varied depending on the seniority of a fellow’s position (Table 3). The more junior associate and technical advisor roles included many objectives at the “understand” and “apply” levels. A few technical advisors and the mid-career technical advisors also included more applied level objectives and also a few at the “create” level. The senior technical advisor roles included a wide range of levels of objectives from “understand” to “create” with most of the objectives at the applied level. Finally, the most senior advisors, “uniquely skilled senior technical advisors” (USSTA) were few in number and had objectives at the “apply” and “analyze” levels.

**Table 3:** Level of Bloom’s Taxonomy for each learning objective by position title*.

	Remember	Understand	Apply	Analyze	Evaluate	Create
Associate III	0	3	4	0	0	0
Associate IV	0	2	4	0	0	1
Technical advisor	0	0	7	0	0	6
Mid-Career Technical Advisor	0	1	20	1	0	3
Mid-Career Technical Advisor II	0	0	3	0	0	0
Senior Technical Advisor	0	12	41	5	4	5
Senior Technical Advisor II	0	1	7	1	0	7
USSTA	0	0	2	1	0	0

*Position titles are in order from lowest or most junior rank first to highest rank last.

### Qualitative exploration of learning objectives within the three most common core competencies

#### Capacity strengthening

US-based fellows had several objective foci related to capacity strengthening. The first was on the development of frameworks to guide and facilitate programming. For example, “develop a framework for demonstrating the impact of formal capacity strengthening activities […].” Further, fellows worked on synthesis of best practices and case studies to support programs and junior colleagues, such as “Cultivate expertise in current health behavior change best practices in order to advise USAID implementing partners and their campaigns.” For those having recently joined offices at headquarters, several objectives focused on expanding their networks within USAID and beyond “in order to identify timely opportunities to contribute analytical inputs that will inform decision-making.”

A few fellows also shifted content areas during their fellowship, and therefore needed to expand their capacity for updated topical knowledge such as changing from an HIV-AIDS focus to working on family planning programs. Publications and sharing knowledge with the scientific community were also an area of focus for US-based fellows with efforts described in the LOs to expand publications and contributions at scientific meetings, such as “Develop an effective and sustainable strategy for being able to produce peer-reviewed publications […].” Finally, providing mentorship to junior colleagues to guide their capacity strengthening priorities and plans was a theme, for example the objective: “Apply mentorship and leadership skills to enhance professional development of mentees in the workplace.”

LMIC-based fellows had a range of foci related to capacity strengthening from curriculum development to mentorship. Topics of objectives focusing on curriculum development included creating or adapting content for online formats, for example: “develop skills and best practices in designing content for e-learning courses.” In addition to teaching and training, coaching of junior colleagues and youth was a focus in objectives, for instance: “develop a skillset in coaching youth and junior colleagues on how to advocate [for causes] to multiple stakeholders.” In addition, preparation and certification to hold leadership positions and advance the role of their organizations was identified among the objectives of several fellows, for instance, “obtain the certifications required to prepare the county to be part of the WHO Tuberculosis Supranational Reference Laboratory Network.”

Developing data analysis skills and expanding proficiency with data analysis software was a frequently noted theme, for example: “Strengthen skills in data analysis and visualization in order to inform national recommendations and program reporting.” These objectives also often are meant to enable the use of data to support achievement of program targets at the national level. Relatedly, some fellows working on infectious diseases such as TB aimed to support or improve national surveillance systems through objectives such as: “develop an understanding of national surveillance systems that aggregate data from all aspects in order to improve reporting.” Gaining up-to-date knowledge from the global experience in order to support colleagues and inform national programs was a recurring theme, i.e. maintaining an “applied understanding of the latest information and best practice related to global TB diagnostics […].” Finally, research expertise such as developing “skills to design and lead an operational research team in order to conduct research in accordance with national priorities” was a focus for several fellows.

#### Communications and interpersonal effectiveness

Fellows had communications-related objectives related to improving language skills. US-based fellows aimed to improve foreign language ability and the synthesis and dissemination of information. Several fellows indicated that they were interested in developing foreign language skills, particularly French and Spanish. Objectives included “develop Spanish language skills to the level of being able to communicate around clinical decision-making in PEPFAR host countries” and “develop foundational skills in French in order to engage directly with Francophone country partners on MEL for nutrition programming.” LMIC-based fellows aimed to improve English skills “in order to communicate more effectively with broader audiences.”

Fellows’ objectives also focused on information dissemination. US-based fellow objectives captured ensuring the use of “key lessons and cases related to key data systems to inform the next generation of data science professionals and projects” and staying “abreast of the latest research and best practices using data analysis in order to inform the design of impactful HIV/AIDS communication programs.” LMIC-based fellows also wanted to work on improving their skills for advocacy and influencing stakeholders through objectives like being able to “effectively navigate communications challenges to advocate for self and the program when working with counterparts, implementing partners and in multilateral settings” and building “analytical skills to better translate concepts between technical professionals and policymakers […].” Finally, fellows also focused on auditing and reporting including gaining skills to “initiate, execute, monitor, evaluate, draft audit reports & carry out improvements to achieve excellence in operation & management of zonal labs” and to “manage relationships between USAID and collaborating organizations in order to support timely MEL reporting and communications to country partners.”

Both US- and LMIC-based fellows also worked on scientific writing skills and productivity including being able to “apply professional and scientific writing skills to support strategic planning and program reporting” and to improve their ability to “formulate a research question, develop and implement a study design, analyze data, and prepare a manuscript for publication.” Fellows from both groups also worked on gaining skills related to incorporating a gender and equity lens into their communications, including being able to “develop a deeper understanding of the health and gender equity domains in order to support the aforementioned, through public health communications efforts” and to “develop foundational knowledge of public health ethics in order to support navigation of ethnical decisions and challenges within TB communications.”

#### Development practice

The STAR core competency development practice focuses on the “ability to collaborate effectively with a variety of development actors from an integrated, intersectoral and global perspective.”(17) Learning objectives related to the development practice competency included a number of general skill building goals related to collaboration, networking, and establishing and sustaining rapport with different stakeholders. For fellows working in the USAID headquarters, these skills included building and enhancing relationships among different branches of USAID, for example, “articulate USAID’s vision, policies, regulations, systems, and cycles and be able to navigate these to meet work objectives” and “effectively utilize USAID processes and strategies to ensure that monitoring, evaluation, and learning relationships and activities within USAID and with external partners are appropriate supported.” US-based fellows also had objectives related to conducting reviews, analyses, or data visualization with USAID and other partners in order to guide programs, such as “Interpret findings from analyses and program experience by convening team members and engaging them to package findings and inform programming.”

STAR fellows based outside the US had development practice-related objectives that emphasized partnership and engagement for the particular programs that they worked with, often with a mentorship component. For example, to “develop a skillset in coaching youth and junior colleagues on how to advocate to multiple stakeholders in order to support the people living with HIV (PLHIV) program.” They also included objectives related to bringing together stakeholders for a particular output or a next step in career progression, including: “develop the skills and network to serve as […] a co-investigator on a national or global research study focused on generating new evidence-based practice around tuberculosis reporting.”

## Discussion

We set out to understand the kind of learning priorities and planned outcomes that global health leaders identified for themselves. We also wanted to reflect on the process of developing and utilizing individualized learning objectives to guide a global health curriculum using the STAR Project as a case example.

Our study has several limitations. The first is that our sample size is small: while we utilized data from all STAR fellows who had onboarded at the time when we began our analysis, the number of participants did not enable us to conduct more in-depth quantitative analyses including statistical significance tests of differences between groups. STAR fellows are also not a random sample of the global health workforce, and so the focus of learning objectives is not necessarily representative of the learning priorities other global health leaders might have. Finally, the STAR learning team invested substantial effort in undertaking joint training on curriculum development—including on the development of high-quality learning objectives—and maintained an ongoing peer review of drafted learning objectives in order to ensure objectives were as consistent as possible. Even with those shared experiences and processes in place, achieving clear, concise, and specific learning objectives remains a challenge.

US-based and LMIC fellows focused on different aspects of programs and were also at different points in their careers. The LMIC-based fellows tended to have a more technical focus on program implementation while those in US-based positions focus more on advising and guiding colleagues by identifying, developing, and packaging global knowledge and supporting programs to utilize it. This has been seen in other programs as well that focus on diverse target audiences [9, 14, 15, 19–23]. The LOs of LMIC-based fellows are largely aimed at developing and then directly applying skills—such as data analysis, language, and communications—in their work. This is partly due to the needs of the fellows and may also be because the STAR team became confident in developing this kind of learning objective and was able to provide relevant activities such as courses and conferences for fellows to achieve such objectives. Levels of LOs along Bloom’s taxonomy varied among participants with no particular trend towards “higher level” learning for more senior fellows; this could be because many STAR participants utilized the fellowship as an opportunity to expand into new areas and acquire new skills.

While the process of developing individualized learning objectives was time-consuming, we found that this approach permitted us to identify learning activities to advance a wide breadth of goals and needs, using a participant-centered approach. Overall in our cohort, we saw a need to identify training resources that focused on program implementation and logistics [12]; a need to develop analytical and scientific writing skills with a view of impactful dissemination and communication [7]; and lastly a focus on de-mystifying the global health ecosystem by strengthening their knowledge of the development practice setting and key actors [7, 12, 24]. We also identified that the needs for LMIC-based fellows and US-based fellows varied significantly, and so our approach needed to be flexible enough to adapt to each group. While there is benefit to north-south exchanges and pooling learning resources in order to make more opportunities available to all, the roles of the individuals can vary greatly, as do their learning needs [8–10, 25, 26]. Training programs also need to be mindful regarding pedagogical approaches that support the training needs of both groups, including the level of learning that is expected and the degree of direct application of learning to practice [14, 16, 27, 28]. This aligns strongly with adult learning theory, of which a key component is engaging learners in their own learning process and ensuring that they can see the value and utility of whatever learning activities they are taking on [5, 29–31].

## Conclusion

Our experience developing and utilizing individualized LOs yielded several important learnings for global health education. First, while the process was labor intensive, we were able to gain insight into the specific needs of different groups of participants, and to also recognize related gaps in terms of what resources were available to actually achieve defined LOs. Common themes in terms of learning objective content included core competencies in development practice, communications, and capacity strengthening. More global health education programs could benefit from investing in tailored processes to ensure that diverse participants define and then meet their own specific learning priorities over the course of a program.

## Supporting information

S1 Annex STAR global health competency domains. (JPG)

S2 Annex Core, skill and content competencies by output. (DOCX)
