# Supplementary figures and images for: Defining and utilizing individualized learning objectives to achieve learning priorities for global health leaders
Source: PLoS One. 2022 Jun 28;17(6):e0270465. doi: 10.1371/journal.pone.0270465 (PMC9239444; doi:10.1371/journal.pone.0270465)

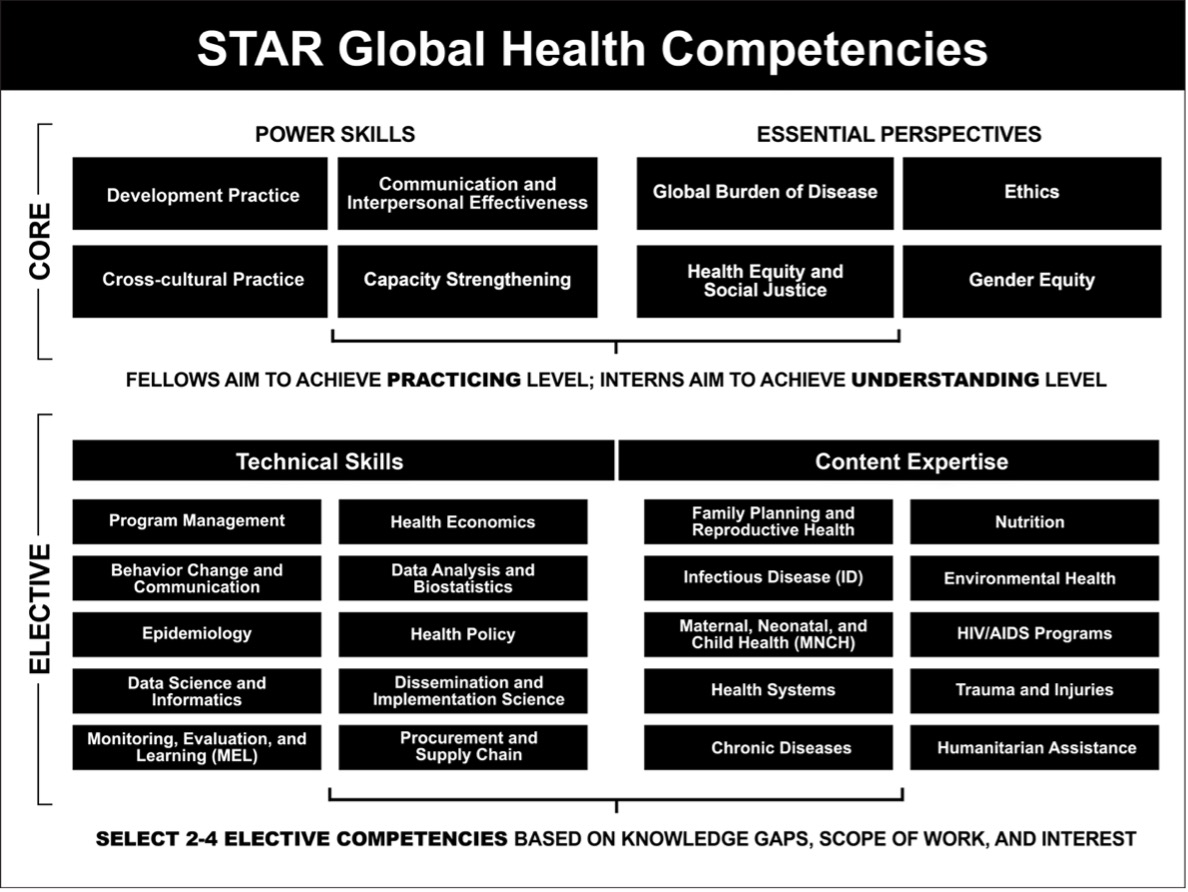

Supplement: S1 Annex — (JPG) [file pone.0270465.s001.jpg]
