# Supplementary material for: Defining and utilizing individualized learning objectives to achieve learning priorities for global health leaders
Source: PLoS One. 2022 Jun 28;17(6):e0270465. doi: 10.1371/journal.pone.0270465 (PMC9239444; doi:10.1371/journal.pone.0270465)
Supplement: S2 Annex — (DOCX) [file pone.0270465.s002.docx]

| **Annex 2: Core, Skill and Content Competencies by Output**   \|  \| **Core competencies** \| \| \| \| \| \| \| \| \| \| --- \| --- \| --- \| --- \| --- \| --- \| --- \| --- \| --- \| --- \| \|  \| Cap  (n = 47) \| Dev  (n = 20) \| Com  (n = 35) \| GBD  (n = 3) \| HE/SJ  (n = 3) \| Cul  (n = 2) \| Eth  (n = 11) \| Gen  (n = 6) \| Total  (N = 127) \| \| Assessment Tool \| 2 (4.3%) \| 0 \| 0 \| 00 \| 0 \| 0 \| 0 \| 0 \| 2 (1.6%) \| \| Analytical Tool \| 2 (4.3%) \| 1 (5.0%) \| 0 \| 0 \| 0 \| 0 \| 0 \| 0 \| 3 (2.4%) \| \| Manuscript \| 0 \| 0 \| 1 (2.9%) \| 0 \| 0 \| 0 \| 0 \| 0 \| 1 (0.8%) \| \| Program Report \| 2 (4.3%) \| 1 (5.0%) \| 1 (2.9%) \| 0 \| 0 \| 0 \| 0 \| 0 \| 4 (3.1%) \| \| Training Module \| 1 (2.1%) \| 0 \| 0 \| 0 \| 0 \| 1 (50.0%) \| 0 \| 0 \| 2 (1.6%) \| \| Data Visualization \| 1 (2.1%) \| 0 \| 1 (2.9%) \| 0 \| 0 \| 0 \| 1 (9.1%) \| 0 \| 3 (2.4%) \| \| Process \| 2 (4.3%) \| 0 \| 0 \| 0 \| 0 \| 0 \| 0 \| 0 \| 2 (1.6%) \| \| Framework \| 2 (4.3%) \| 0 \| 1 (2.9%) \| 0 \| 0 \| 0 \| 2 (18.2%) \| 1 (16.7%) \| 6 (4.7%) \| \| Data System \| 1 (2.1%) \| 0 \| 0 \| 0 \| 1 (33.3%) \| 0 \| 0 \| 0 \| 2 (1.6%) \| \| Statistics/Analysis \| 1 (2.1%) \| 0 \| 1 (2.9%) \| 0 \| 0 \| 0 \| 0 \| 0 \| 2 (1.6%) \| \| Guidelines \| 1 (2.1%) \| 0 \| 1 (2.9%) \| 0 \| 0 \| 0 \| 0 \| 0 \| 2 (1.6%) \| \| Advising \| 10 \| 4 (20.0%) \| 5 (14.3%) \| 1 (33.3%) \| 0 \| 0 \| 3 (27.3%) \| 2 (33.3%) \| 25 (19.7%) \| \| Communications Maternal \| 2 (4.3%) \| 0 \| 2 (5.7%) \| 0 \| 0 \| 0 \| 0 \| 1 (16.7%) \| 5 (29.4%) \| \| Knowledge Management \| 4 (8.5%) \| 0 \| 0 \| 0 \| 0 \| 0 \| 0 \| 0 \| 4 (3.1%) \| \| Mentor/Supervise \| 3 (6.4%) \| 2 (10.0%) \| 4 (11.4%) \| 0 \| 0 \| 0 \| 0 \| 0 \| 9 (7.1%) \| \| Systematic Review \| 0 \| 1 (5.0%) \| 0 \| 0 \| 0 \| 0 \| 0 \| 0 \| 1 (0.8%) \| | | | | | | | | | | | |
| --- | --- | --- | --- | --- | --- | --- | --- | --- | --- | --- | --- | --- | --- | --- | --- | --- | --- | --- | --- | --- | --- | --- | --- | --- | --- | --- | --- | --- | --- | --- | --- | --- | --- | --- | --- | --- | --- | --- | --- | --- | --- | --- | --- | --- | --- | --- | --- | --- | --- | --- | --- | --- | --- | --- | --- | --- | --- | --- | --- | --- | --- | --- | --- | --- | --- | --- | --- | --- | --- | --- | --- | --- | --- | --- | --- | --- | --- | --- | --- | --- | --- | --- | --- | --- | --- | --- | --- | --- | --- | --- | --- | --- | --- | --- | --- | --- | --- | --- | --- | --- | --- | --- | --- | --- | --- | --- | --- | --- | --- | --- | --- | --- | --- | --- | --- | --- | --- | --- | --- | --- | --- | --- | --- | --- | --- | --- | --- | --- | --- | --- | --- | --- | --- | --- | --- | --- | --- | --- | --- | --- | --- | --- | --- | --- | --- | --- | --- | --- | --- | --- | --- | --- | --- | --- | --- | --- | --- | --- | --- | --- | --- | --- | --- | --- | --- | --- | --- | --- | --- | --- | --- | --- | --- | --- | --- | --- | --- | --- | --- | --- | --- | --- | --- | --- | --- | --- | --- | --- | --- | --- | --- |
|  | **Skill competencies** | | | | | | | | | | |
|  | BCC  (n = 8) | Data Sci  (n = 15) | Epi  (n = 12) | DI  (n = 8) | MEL  (n = 14) | Econ  (n = 4) | Supply Chain  (n = 12) | Pol  (n = 6) | Stats  (n = 23) | PM  (n = 20) | Total  (N = 84) |
| Assessment Tool | 0 | 0 | 0 | 0 | 1 (33.3%) | 0 | 2 (66.7%) | 0 | 0 | 0 | 3 |
| Analytical Tool | 0 | 2 (18.2%) | 1 (9.1%) | 0 | 1 (9.1%) | 1 (9.1%) | 2 (18.2%) | 2 (18.2%) | 1 (9.1%) | 1 (9.1%) | 11 |
| Manuscript | 0 | 1 (33.3%) | 0 | 0 | 0 | 0 | 0 | 0 | 1 (33.3%) | 1 (33.3%) | 3 |
| Program Report | 0 | 1(16.7%) | 1 (16.7%) | 1 (16.7%) | 1 (16.7%) | 0 | 1 (16.7%) | 0 | 1 (16.7%) | 0 | 6 |
| Training Module | 0 | 1 (50.0%) | 0 | 0 | 0 | 0 | 0 | 0 | 1 (50.0%) | 0 | 2 |
| Data Visualization | 0 | 0 | 1 (16.7%) | 0 | 0 | 0 | 0 | 1 (16.7%) | 3 (50.0%) | 1 (16.7%) | 6 |
| Process | 0 | 0 | 0 | 0 | 1 (25.0%) | 0 | 0 | 0 | 0 | 3 (75.0%) | 4 |
| Framework | 1 (25.0%) | 0 | 1 (25.0%) | 0 | 1 (25.0%) | 0 | 1 (25.0%) | 0 | 0 | 0 | 4 |
| Data System | 0 | 2 (66.7%) | 0 | 0 | 0 | 0 | 0 | 0 | 1 (33.3%) | 0 | 3 |
| Statistics/Analysis | 0 | 0 | 0 | 0 | 0 | 0 | 0 | 0 | 2 (100.0%) | 0 | 2 |
| Guidelines | 0 | 0 | 0 | 2 (66.7%) | 0 | 0 | 0 | 0 | 0 | 1 (33.3%) | 3 |
| Advising | 1 (3.6%) | 4 (14.3%) | 3 (10.7%) | 3 (10.7%) | 2 (7.1%) | 2 (7.1%) | 1 (3.6%) | 3 (10.7%) | 3 (10.7%) | 6 (21.4%) | 28 |
| Communication Maternal | 0 | 0 | 0 | 1 (100.0%) | 0 | 0 | 0 | 0 | 0 | 0 | 1 |
| Knowledge Management | 1 (20.0%) | 0 | 0 | 2 (40.0%) | 1 (20.0%) | 0 | 1 (20.0%) | 0 | 0 | 0 | 5 |
| Mentor/Supervise | 0 | 0 | 0 | 0 | 1 (50.0%) | 0 | 1 (50.0%) | 0 | 0 | 0 | 2 |
| Systematic Review | 0 | 0 | 1 (100.0%) | 0 | 0 | 0 | 0 | 0 | 0 | 0 | 1 |

|  | **Content Competencies*** | | | | |
| --- | --- | --- | --- | --- | --- |
|  | Nut  (n = 4) | FPRH  (n = 4) | ID  (n = 54) | HIV  (n = 11) | Total  (N = 43) |
| Assessment Tool | 0 | 1 (50.0%) | 1 (50.0%) | 0 | 2 |
| Analytical Tool | 0 | 0 | 4 (80.0%) | 1 (20.0%) | 5 |
| Manuscript | 0 | 0 | 1 (100.0%) | 0 | 1 |
| Program Report | 1 (50.0%) | 0 | 1 (50.0%) | 0 | 2 |
| Training Module | 0 | 0 | 0 | 0 | 0 |
| Data Visualization | 0 | 0 | 0 | 0 | 0 |
| Process | 0 | 0 | 2 (66.7%) | 1 (33.3%) | 3 |
| Framework | 0 | 0 | 1 (100.0% | 0 | 1 |
| Data System | 0 | 0 | 3 (100.0%) | 0 | 3 |
| Statistics/Analysis | 0 | 0 | 1 (100.0%) | 0 | 1 |
| Guidelines | 0 | 0 | 1 (100.0%) | 0 | 1 |
| Advising | 0 | 0 | 15 (83.3%) | 3 (16.7%) | 18 |
| Communication Maternal | 0 | 0 | 1 (100.0%) | 0 | 1 |
| Knowledge Management | 1 (33.3%) | 1 (33.3%) | 1 (33.3%) | 0 | 3 |
| Mentor/Supervise | 0 | 0 | 1 (50.0%) | 1 (50.0%) | 2 |
| Systematic Review | 0 | 0 | 0 | 0 | 0 |

*Additional content competencies in the STAR framework that were never selected for participant LOs: Technology and Innovation, Environmental Health, Maternal, Neonatal, and Child Health, Health Systems, Chronic Diseases.
